# Professionalism and e‐Professionalism From Dentists’ Perspective: A Multicenter Cross‐Sectional Study

**DOI:** 10.1155/ijod/5856512

**Published:** 2026-02-26

**Authors:** Rasha A. Alamoush, Razan Alaqeely, Suhad J. Al-Nasrawi, Julfikar Haider, Manar Mar’i, Raneem Alkhader, Mahmoud K. Al-Omiri

**Affiliations:** ^1^ Department of Fixed and Removable Prosthodontics, School of Dentistry, The University of Jordan, Queen Rania Street, Amman, 11942, Jordan, ju.edu.jo; ^2^ Department of Dentistry, The University of Jordan Hospital, Amman, 11942, Jordan; ^3^ Department of Periodontics and Community Dentistry, College of Dentistry, King Saud University, Riyadh, Saudi Arabia, ksu.edu.sa; ^4^ Department of Conservative Dentistry, Faculty of Dentistry, University of Kufa, Najaf, Iraq, uokufa.edu.iq; ^5^ Department of Engineering, Manchester Metropolitan University, Manchester, UK, mmu.ac.uk

**Keywords:** attitude, dentists’ perception, multicenter, professionalism, quality of life, sociodemographic factors

## Abstract

**Objectives:**

This study aimed to investigate professionalism and e‐professionalism from the perspectives of students and dentists in multiregional countries (Saudi, Iraq, and Jordan).

**Methods:**

Data were collected using a self‐administered questionnaire that investigated professionalism from students’ and dentists’ perspectives in multiregional countries (Saudi, Iraq, and Jordan). Participants were asked specific questions regarding sociodemographic factors, including gender, region, age, educational level, years of experience, and work sector, and questions about professionalism, including professional attitude and behavior, ethics and jurisprudence, consciousness, communication, and interpersonal skills, quality of life and personal satisfaction, and e‐professionalism. The questionnaire was scored with participants’ answers following an agreement scale, from strongly agree to strongly disagree. The data from 292 participants (91 males and 201 females) were collected and analyzed using SPSS computer software. Statistical significance was considered at 95% confidence intervals and a two‐tailed *α* of 0.05.

**Results:**

The results showed that participants from Jordan and Saudi Arabia were more aware of the meaning and aspects of professionalism and thought that professionalism should be included as part of the undergraduate curriculum than were participants from Iraq (*p*  < 0.001), with more participants from Saudi Arabia taking educational or training courses about professionalism than participants from Jordan or Iraq (*p*  < 0.001). No significant differences were found between genders regarding awareness of the meaning and aspects of professionalism. Furthermore, significant differences were found between different levels of education regarding awareness of the meaning and aspects of professionalism (*p*  < 0.001). Older participants were more aware of the meaning and aspects of professionalism than younger participants were (*p*  < 0.001). Additionally, no relationship was found between the work sector and awareness of the meaning and aspects of professionalism. The Jordanian participants scored more than the Saudi and Iraqi participants on each item, as did the professionalism subscale (*p*  < 0.05). Moreover, Saudi participants scored higher than Iraqi participants in all significantly different item responses except for questions 29, 30, 33, 38, and 39, as well as in all significantly different professionalism subscale scores except for the quality of life and personal satisfaction subscale scores (*p*  < 0.05).

**Conclusions:**

The regional differences in awareness and attitudes toward professionalism among dental professionals and students from Jordan, Iraq, and Saudi Arabia were highlighted, and notably, Jordanian and Saudi participants demonstrated higher levels of professionalism than the participants from Iraq. Factors such as age, gender, work sector, and nationality significantly influenced professionalism, with older professionals, females, and those in the private sector generally scoring higher. The study also highlighted uniformly high communication skills across regions and variations in e‐professionalism scores. It was also emphasized that sociocultural factors contributed to improved quality of life and personal satisfaction in Jordan and Iraq.

## 1. Introduction

Professionalism has been recognized as a vital element of dental practice. It could be defined as the adapting of high, technical, intellectual, and moral qualities, performance, and abilities in providing service to attended patients and the community. However, it is related to a set of values, relationships, and behaviors that underpin the public trust in dentists [1, 2].

Many dental organizations and associations consider academic integrity a crucial element of professionalism because they are closely related [3, 4]. Thus, training in professionalism is greatly relevant for prospective dentists. Dental schools are accountable for providing academic lessons that include both precise technical experiences and transversal competencies, ensuring the development of certain attitudes, ethical behaviors, and moral values among dental students [2, 3, 5–8]. There are six comprehensive components of professionalism that have been tainted by the American Board of Internal Medicine, including integrity, altruism, duty, excellence, accountability, honor, and respect for others [4], reinforcing the public’s trust in dental professionals. Therefore, adequate knowledge, practical experiences, and communication skills can assist in the development of a professional’s specialized awareness, knowledge, and skills [9, 10]. A considerable number of previously published research has shown an optimistic understanding of professionalism among medical students and trainees [11, 12].

Professionalism is considered a vital aspect for dentists, concerning patient confidentiality and communication skills with persons in the surrounding treatment field, which are difficult to measure and teach [13]. Thus, it is important to include it throughout the educational period [14]. This will offer an enduring foundation for teaching, learning, assessing, and most importantly, obtaining professionalism for future dentists and dental practices [15]. To create a professional attitude among dentists, logically, it is important to recognize the level of student awareness and their perception of what is professional and what is not [16].

Cultural and political variations have a great influence on dentistry, so professionalism among healthcare providers might vary for different geographical locations and cultures. Numerous cultural differences exist when defining professionalism [17–19]. As a result of the absence of an acceptable theoretical setting for professionalism [20], professionals in the Asian community may not be accepted as professionals by patients and society in African society [18]. Nevertheless, efforts are being made to make professionalism a fundamental component of the dental and healthcare professional education system [21]. The increased global application of social media and networking among healthcare workers has enhanced professional networking, collaboration, interactive communication, education, and training [22]. This has a great impact on the traditional concepts of ethics, privacy, and medical professionalism [22, 23]. Subsequently, the term “e‐professionalism” has developed, representing a new form of professionalism. Professional identity, attitude, and behavior are manifested through online platforms and digital media [24]. Digital technology greatly influences the development of dental professionals and training [25].

Many studies have explored professionalism; however, no study has explored the impact of various social factors—such as gender, educational level, geographic region, and work sector—on the development of professional attitudes and behaviors among dental students and dentists in the Middle East. Further, limited research has assessed the combined impact of traditional professionalism and e‐professionalism among dental students and dentists. Therefore, this study aimed to investigate the levels of professionalism and e‐professionalism among dental health providers across Saudi Arabia, Iraq, and Jordan and to analyze how social, economic, and educational factors contribute to variations in professional behavior. The null hypothesis is stated as there is no impact of social, economic, educational, or geographic factors on professional behavior and e‐professionalism.

## 2. Methods

### 2.1. Ethical Approval

The research protocol was approved by the Ethical Committee of the Faculty of Dentistry of the University of Jordan (Reference: 30‐2023) according to the World Medical Declaration of Helsinki. All the participants were informed regarding the aim and objectives of the questionnaire and agreed to fill out the form voluntarily. No personal information of participants or students was exposed or traced back to individuals as the questionnaire was anonymized and the appropriate protocol was followed so personal information was securely stored.

### 2.2. Study Group and Design

This is a cross‐sectional (online) questionnaire that investigates professionalism and e‐professionalism from students’ and dentists’ perspectives in multiregional countries (Saudi, Iraq, and Jordan).

The data were collected using a self‐administered questionnaire that investigated professionalism from students’ and dentists’ perspectives in multiregional countries (Saudi, Iraq, and Jordan). The study has four sections. Participants were asked specific questions regarding sociodemographic factors, including gender, region, age, educational level, years of experience, and work sector, and questions about professionalism, including professional attitude and behavior, ethics and jurisprudence, consciousness, communication, and interpersonal skills, quality of life and personal satisfaction, and e‐professionalism.

The questionnaire was adapted from validated instruments used in previous studies on professionalism and e‐professionalism in healthcare education [14, 25–30]. Before full administration, the questionnaire was pilot‐tested with a sample of 15 dental students and practitioners to assess clarity, relevance, and reliability. Minor adjustments were made based on participant feedback to improve comprehension. The Cronbach’s alpha score for the questionnaire was 0.904, indicating adequate reliability during the pilot study. Subsequently, the survey was administered to the participants in the main research, and data collection commenced. The Cronbach’s alpha score for the questionnaire in the main study was 0.899, also indicating adequate reliability.

The objective of this study was to determine the level of professionalism of dental students and dentists. The questionnaire was scored by participants’ answers according to an agreement scale (Likert scale), from strongly disagreed (score 1) to strongly agreed (score 5).

### 2.3. Data Collection Method

Participants were recruited through online invitations disseminated via professional dental associations’ mailing lists, university platforms, and social media groups targeting dental students and practitioners in the three countries. Inclusion criteria included individuals currently enrolled in dental education programs or practicing as licensed dentists. No exclusion criteria were applied aside from incomplete questionnaire submissions; however, all submitted questionnaires were complete and thus included in the analysis. No questionnaires with missing or incomplete data were included in the final analysis. All 292 returned questionnaires were fully completed; thus, no imputation or exclusion for missing data was necessary. The estimated sample size was 204 participants. Further participants were invited and recruited to secure the required sample size. *Four hundred* participants were invited to participate in the study, and 292 responded (*73%* response rate). The data from 292 participants (91 males and 201 females) were collected and analyzed. The data included 107 participants from Jordan (36.6%), 93 from Iraq (31.8%), and 92 from Saudi Arabia (31.5%). The participants’ ages ranged between 18 and 70 years. Table 1 presents a summary of the demographic data of the study population.

**Table 1:** Distribution of demographic and general variables among the total study participants as well as in each country (*n* = 292).

Categorical nominal variables		All sample (*n* = 292)	Jordan (*n* = 107)	Iraq (*n* = 93)	Saudi Arabia (*n* = 92)
		*N* (%)	*N* (%)	*N* (%)	*N* (%)
Gender	Male	91 (31.2)	23 (21.5)	26 (28.0)	42 (45.7)
	Female	201 (68.8)	84 (78.5)	67 (72.0)	50 (54.3)
Age	18–28 years	158 (54.1)	73 (68.2)	58 (62.4)	27 (29.3)
	29–39 years	81 (27.7)	22 (20.6)	19 (20.4)	40 (43.5)
	40–49 years	25 (8.6)	5 (4.7)	9 (9.7)	11 (12.0)
	50–60 years	19 (6.5)	6 (5.6)	3 (3.2)	10 (10.9)
	Above 60 years	9 (3.1)	1 (0.9)	4 (4.3)	4 (4.3)
Level of education	Undergraduate	68 (23.3)	11 (10.3)	51 (54.8)	6 (6.5)
	BDS	91 (31.2)	64 (59.8)	4 (4.3)	23 (25.0)
	MSc/residency	93 (31.8)	26 (24.3)	29 (31.2)	38 (41.3)
	PhD	40 (13.7)	6 (5.6)	9 (9.7)	25 (27.2)
Work sector	Private sector	65 (22.3)	48 (44.9)	1 (1.1)	16 (17.4)
	Public sector	48 (16.4)	11 (10.3)	5 (5.4)	32 (34.8)
	Academic sector	179 (61.3)	48 (44.9)	87 (93.5)	44 (47.8)
Experience	Less than 1 year	80 (27.4)	30 (28.0)	38 (40.9)	12 (13.0)
	1–5 years	95 (32.5)	46 (43.0)	21 (22.6)	28 (30.4)
	6–10 years	33 (11.3)	8 (7.5)	10 (10.8)	15 (16.3)
	Above 10 years	84 (28.8)	23 (21.5)	24 (25.8)	37 (40.2)
Preferred application to communicate with patients	Facebook	22 (7.5)	12 (11.2)	10 (10.8)	0 (0.0)
	Instagram	63 (21.6)	24 (22.4)	24 (25.8)	15 (16.3)
	WhatsApp	171 (58.6)	57 (53.3)	50 (53.8)	64 (69.6)
	Others	36 (12.3)	14 (13.1)	9 (9.7)	13 (14.1)
Spent time on social media	0–2 h	70 (24.0)	19 (17.8)	25 (26.9)	26 (28.3)
	2–4 h	111 (38.0)	45 (42.1)	30 (32.3)	36 (39.1)
	4–6 h	86 (29.5)	35 (32.7)	28 (30.1)	23 (25.0)
	Above 6 h	25 (8.6)	8 (7.5)	10 (10.8)	7 (7.6)
Do you interact with clinical staff/lecturers via social media?	No	64 (21.9)	15 (14.0)	18 (19.4)	31 (33.7)
	Yes	228 (78.1)	92 (86.0)	75 (80.6)	61 (66.3)
Have you discussed clinical cases on social media?	No	115 (39.4)	36 (33.6)	30 (32.3)	49 (53.3)
	Yes	177(60.6)	71 (66.4)	63 (67.7)	43 (46.7)
Do use closed/private or public/open groups to discuss patients	Private groups	150 (51.4)	60 (56.1)	51 (54.8)	39 (42.4)
	Public groups	49 (16.8)	18 (16.8)	21 (22.6)	10 (10.9)
	None	93 (31.8)	29 (27.1)	21 (22.6)	43 (46.7)
Have you added or accepted patients on your social media accounts?	No	152 (52.1)	62 (57.9)	30 (32.3)	60 (65.2)
	Yes	140 (47.9)	45 (42.1)	63 (67.7)	32 (34.8)

### 2.4. Statistical Analysis

SPSS software (IBM SPSS Statistics v20.0; IBM Corp., USA) was used to perform the data analysis. The study variables were tested for a normal distribution; then, the variables were summarized accordingly. The continuous variables (professionalism total and subscale scores) were not normally distributed; thus, the median, interquartile range, and range are presented for these variables. The categorical variables are presented as frequencies and percentages.

Comparisons between groups based on country and work sector were performed utilizing the Kruskal–Wallis test. The Mann–Whitney *U* test was used to compare two groups and to identify differences between sexes regarding categorical and nonnormally distributed continuous variables. Spearman’s Rho rank correlations were utilized to assess the relationships between total and subscale scores of professional well‐being and age and level of education.

Multiple stepwise linear regression analysis was utilized to examine the odds of total professionalism and subscale scores utilizing age, gender, level of education, country, and work sector variables. Statistical significance was considered at 95% confidence intervals and a two‐tailed *α* of 0.05 throughout this investigation.

Given the exploratory nature of this study and the multiple independent variables considered, stepwise linear regression was selected to identify the most significant predictors of professionalism scores. Prior to regression, all variables were tested for normality using the Shapiro–Wilk test. Although some variables with nonnormal distributions underwent linear regression analysis, the residuals were normally distributed as tested via the skewness test with skewness values of the standardized and unstandardized residuals of the professionalism scores ranged between −3.29 and +3.29, which confirms normal distribution for sample sizes between 50 and 300. In addition, the normal P–P plots showed linear trends between the standardized residual values (ZResid values) and predicted values (ZPred values). Additionally, homoscedasticity was tested via residual scatter plots which showed that the standardized residuals were scattered between −3 and 3 values without the presence of trends of values within the scatter. Furthermore, independence of residuals and absence of multicollinearity were assessed through, Durbin–Watson test (value ranged between 2.08 and 2.206) and variance inflation factor (VIF) values (VIF value was 1 for all stepwise regressions), respectively, confirming the suitability of the regression models.

A priori power analysis utilizing the linear multiple regression test of the F test family with a two‐tailed *α* probability error of 0.05, a statistical power (1–β) of 0.95, and an effect size of 0.1 was carried out to calculate the size of the study sample for this investigation using 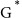
Power software (version 3.1.9.7; Heinrich‐Heine University).

## 3. Results

Table 2 shows that participants from Jordan and Saudi Arabia were more aware of the meaning and aspects of professionalism and thought that professionalism should be included as part of the undergraduate curriculum than participants from Iraq (*p*  < 0.001, Table 2). Additionally, more participants from Saudi Arabia than participants from Jordan or Iraq had educational or training courses about professionalism (*p*  < 0.001, Table 2). In addition, most participants were willing to take professional courses regardless of the country (*p* = 0.206, Table 2).

**Table 2:** Comparison between countries regarding awareness of the meaning and aspects of professionalism, whether to include professionalism in the undergraduate curriculum, and whether to take or be willing to take professional courses (*n* = 292).

Item	Response	Country (*n*)			Cramer’s *V*	Kruskal–Wallis test	
		Jordan	Iraq	Saudi		*X* ^2^ (df = 2)	*p*
Are you aware of professionalism meaning and aspects?	Extremely not aware	0	1	1	0.334	46.330	<0.001
	Slightly not aware	4	6	1			
	Neutral	17	44	5			
	Slightly aware	43	20	29			
	Extremely aware	43	22	56			
Do you think professionalism should be included as part of undergraduate curriculum?	Strongly disagree	1	0	0	0.315	50.144	<0.001
	Slightly disagree	1	4	1			
	Neutral	10	34	4			
	Slightly agree	22	24	16			
	Strongly agree	73	31	71			
Have you had any educational or training courses about professionalism?	No	86	70	42	0.325	30.695	<0.001
	Yes	21	23	50			
Are you willing to take such courses?	No	13	5	11	0.104	3.157	0.206
	Yes	94	88	81			

Item	Compared groups	Mann–Whitney *U* test					
		Mann–Whitney *U*			*Z*‐statistic		*P*
Are you aware of professionalism meaning and aspects?	Jordan vs. Iraq	3293.0			−4.330		<0.001
	Jordan vs. Saudi	3759.0			−3.155		0.002
	Iraq vs. Saudi	2045.5			−6.506		<0.001
Do you think professionalism should be included as part of undergraduate curriculum?	Jordan vs. Iraq	2981.5			−5.340		<0.001
	Jordan vs. Saudi	4441.0			−1.516		0.130
	Iraq vs. Saudi	2167.5			−6.425		<0.001
Have you had any educational or training courses about professionalism?	Jordan vs. Iraq	4721.5			−0.867		0.386
	Jordan vs. Saudi	3213.0			−5.085		<0.001
	Iraq vs. Saudi	3011.0			−4.110		<0.001
Are you willing to take such courses?	Jordan vs. Iraq	4638.5			−1.665		0.096
	Jordan vs. Saudi	4912.5			−0.042		0.967
	Iraq vs. Saudi	3996.5			−1.588		0.112

*Note:* Cramer’s V, symmetric measure of association (effect size); *X* ^2^, Chi square statistic; Mann–Whitney *U*, Mann–Whitney *U* statistic; *p*, probability value.

Abbreviation: df, degree of freedom.

The results showed no significant differences were found between genders regarding awareness of the meaning and aspects of professionalism (Mann–Whitney *U* = 8594.0, *Z* = −0.877, and *p* = 0.381), whether to include professionalism in the undergraduate curriculum (Mann–Whitney *U* = 8720.0, *Z* = −0.725, and *p* = 0.468) or willingness to take professional courses (Mann–Whitney *U* = 8859.0, *Z* = −0.828, and *p* = 0.408). However, females took more courses regarding professionalism than males did (Mann–Whitney *U* = 7582.5, *Z* = −2.890, and *p* = 0.004).

Furthermore, significant differences were found between different levels of education regarding awareness of the meaning and aspects of professionalism (Kruskal–Wallis test: *X* ^2^ = 24.284, df = 3, and *p*  < 0.001) as well as whether to include professionalism in the undergraduate curriculum (Kruskal–Wallis test: *X* ^2^ = 9.054, df = 3, and *p* = 0.029). The undergraduate students were less aware of the meaning and aspects of professionalism than were the interns and BDS participants (Mann–Whitney *U* = 2192.0, *Z* = –3.312, and *p* = 0.001), postgraduate and MSc participants (Mann–Whitney *U* = 2179.5, *Z* = –3.537, and *p*  < 0.001), and PhD participants (Mann–Whitney *U* = 704.5, *Z* = −4.397, and *p*  < 0.001). Additionally, the undergraduate students thought that less professionalism should be included in the undergraduate curriculum than did the interns and BDS participants (Mann–Whitney *U* = 2486.5, *Z* = −2.348, and *p* = 0.019), postgraduate and MSc participants (Mann–Whitney *U* = 2529.0, *Z* = −2.425, and *p* = 0.015), and PhD participants (Mann–Whitney *U* = 1021.5, *Z* = −2.382, and *p* = 0.017).

Moreover, older participants were more aware of the meaning and aspects of professionalism than younger participants were (Spearman’s rho = 0.222 and *p*  < 0.001). Moreover, no significant relationships were found between age and participants’ opinions regarding whether to include professionalism in the undergraduate curriculum (Spearman’s rho = 0.077 and *p* = 0.191) or whether they took (Spearman’s rho = 0.066 and *p* = 0.263) or were willing to take (Spearman’s rho = −0.065 and *p* = 0.267) professional courses. Additionally, no relationship was found between the work sector and awareness of the meaning and aspects of professionalism (Kruskall–Wallis test: *X* ^2^ = 1.824, df = 2, and *p* = 0.402) or whether they took (*X* ^2^ = 3.807, df = 2, and *p* = 0.149) or were willing to take (*X* ^2^ = 1.443, df = 2, and *p* = 0.486) professional courses. Moreover, the work sector was significantly related to the belief that professionalism should be included in the undergraduate curriculum (*X* ^2^ = 7.111, df = 2, and *p* = 0.029). The participants from the private sector thought less that professionalism should be included in the undergraduate curriculum than the academic participants (Mann–Whitney *U* = 4815.0, *Z* = −2.323, and *p* = 0.020).

**Figure 1:**
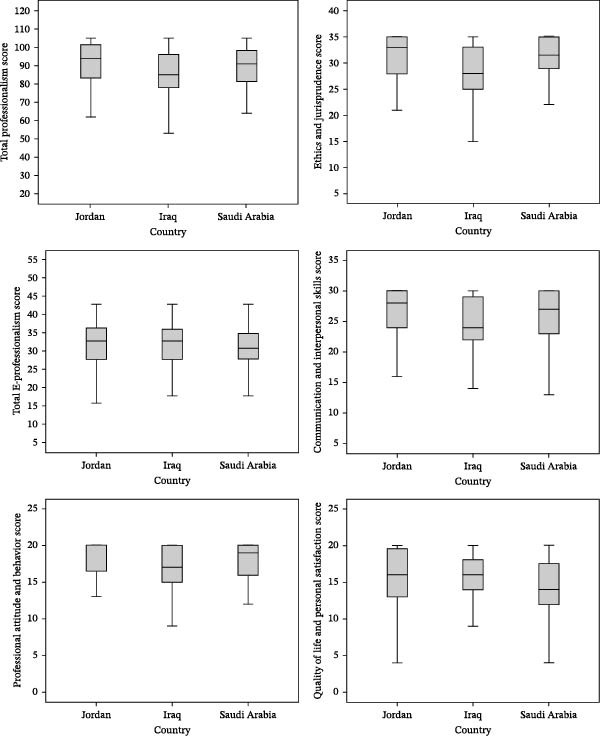
Boxplot graphs for the distribution of total and subscale professionalism scores among the study participants by country.

Table 3 demonstrates the distribution of descriptive statistics for total and subscale professionalism scores based on country, gender, and work sector. Figure 1 presents the boxplot graphs for the distribution of total and subscale professionalism scores based on country.

**Table 3:** Distribution of descriptive statistics for total and subscale professionalism scores based on country, gender, and work sector among the study population (*n* = 292).

Score	Variable			Mean (SD)	Med	Mn–Mx	IQR
Total e‐professionalism score	All sample			33.71 (7.181)	35.00	9–45	8
	Country	Jordan		33.93 (7.252)	35.00	9–45	9
		Iraq		34.00 (7.091)	35.00	9–45	8
		Saudi		33.16 (7.234)	33.00	9–45	7
	Gender	Male		33.10 (7.703)	34.00	9–45	8
		Female		33.99 (6.934)	35.00	9–45	8
	Work sector	Private		33.42 (6.531)	33.00	11–45	8
		Public		33.92 (6.863)	34.50	9–45	7
		Academic		33.76 (7.515)	35.00	9–45	8
Professional attitude and behavior score	All sample			17.20 (3.950)	19.00	4–20	4
	Country	Jordan		17.34 (4.274)	20.00	4–20	4
		Iraq		16.77 (3.609)	17.00	5–20	5
		Saudi		17.46 (3.895)	19.00	4–20	4
	Gender	Male		16.75 (4.231)	18.00	4–20	4
		Female		17.40 (3.809)	19.00	4–20	4
	Work sector	Private		17.43 (3.812)	19.00	4–20	4
		Public		17.42 (3.988)	19.50	4–20	4
		Academic		17.05 (4.003)	18.00	4–20	4
Ethics and jurisprudence score	All sample			29.49 (6.429)	31.00	7–35	8
	Country	Jordan		29.93 (6.930)	33.00	8–35	7
		Iraq		28.34 (5.692)	28.00	9–35	8
		Saudi		30.12 (6.443)	31.50	7–35	6
	Gender	Male		28.55 (6.748)	30.00	7–35	6
		Female		29.91 (6.251)	32.00	7–35	8
	Work sector	Private		30.75 (5.353)	32.00	10–35	6
		Public		29.19 (6.699)	31.00	7–35	9
		Academic		29.11 (6.683)	31.00	7–35	8
Communication and interpersonal skills score	All sample			24.95 (5.675)	26.00	6– 30	7
	Country	Jordan		25.44 (5.978)	28.00	6–30	6
		Iraq		24.16 (4.906)	24.00	6–30	7
		Saudi		25.16 (6.006)	27.00	6–30	7
	Gender	Male		24.40 (6.016)	26.00	6–30	7
		Female		25.19 (5.512)	26.00	6–30	7
	Work sector	Private		25.78 (4.836)	27.00	8–30	6
		Public		24.69 (6.375)	26.50	6–30	8
		Academic		24.71 (5.761)	25.00	6–30	7
Quality of life and personal satisfaction score	All sample			15.06 (4.172)	16.00	4–20	6
	Country	Jordan		15.31 (4.221)	16.00	4–20	7
		Iraq		15.66 (3.778)	16.00	4–20	4
		Saudi		14.17 (4.387)	14.00	4–20	6
	Gender	Male		14.70 (4.753)	16.00	4–20	7
		Female		15.22 (3.883)	16.00	4–20	6
	Work sector	Private		15.80 (3.898)	16.00	6–20	7
		Public		14.81 (4.170)	15.50	4–20	7
		Academic		14.86 (4.259)	16.00	4–20	5
Total professionalism score	All sample			86.69 (18.276)	91.00	21–105	19
	Country		Jordan	88.02 (19.555)	94.00	23–105	18
			Iraq	84.94 (16.517)	85.00	24–105	19
			Saudi	86.91 (18.482)	91.00	21–105	17
	Gender		Male	84.40 (19.834)	90.00	21–105	21
			Female	87.73 (17.478)	91.00	22–105	19
	Work sector		Private	89.77 (15.634)	93.00	28–105	16
			Public	86.10 (19.217)	92.50	21–105	21
			Academic	85.73 (18.872)	89.00	21–105	19

*Note:* SE, standard error of the mean; Mn–Mx, maximum and minimum scores.

Abbreviations: CI, confidence intervals; IQR, interquartile range; Med, median; SD, standard deviation.

Table 4 includes the comparison of the individual professional item responses and total/subscale professionalism scores based on the country of the study population. The Jordanian participants scored more than did the Saudi and Iraqi participants on each item, as did the professionalism subscale (*p*  < 0.05, Table 4). Moreover, Saudi participants scored higher than Iraqi participants in all significantly different item responses except for Questions 29, 30, 33, 38, and 39, as well as in all significantly different professionalism subscale scores except for the quality of life and personal satisfaction subscale scores (*p*  < 0.05, Table 4).

**Table 4:** Comparison of individual professional item responses and total/subscale scores between countries in the study population (*n* = 292).

Professionalism items and scale scores	Kruskal–Wallis test	Jordan vs. Iraq^a^		Jordan vs. Saudi^a^		Iraq vs. Saudi^b^	
	*X* ^2^ (*p*)	MWU	*Z* (*p*)	MWU	*Z* (*p*)	MWU	Z (*p*)
Total professionalism score	6.654 (0.036)	3974.0	−2.457 (0.014)	4399.5	−1.293 (0.196)	3737.0	−1.487 (0.137)
Q12. Professionalism involves demonstrating appropriate caring behavior and a willingness to assist patients	12.883 (0.002)	3976.5	−2.752 (0.006)	4732.0	−0.580 (0.562)	3217.5	−3.296 (0.001)
Q13. Professionalism involves demonstrating respectful and professional behavior toward the dental team members	13.714 (0.001)	4143.5	−2.355 (0.019)	4504.0	−1.358 (0.175)	3177.0	−3.593 (<0.001)
Q14. Professionalism involves understanding the significance of health concerning occupational hazards and their impact	1.590 (0.452)	4532.0	−1.223 (0.221)	4841.0	−0.231 (0.817)	3988.0	−0.891 (0.373)
Q15. Professionalism involves the ability to manage dental practice, patient communication, and the financial aspects of practice	1.485 (0.476)	4542.5	−1.184 (0.236)	4626.0	−0.825 (0.409)	4166.0	−0.338 (0.735)
Professional attitude and behavior score	6.332 (0.042)	4072.5	−2.332 (0.020)	4680.0	−0.640 (0.522)	3613.0	−1.898 (0.058)
Q16. Professionalism involves awareness of patients’ rights, especially confidentiality, patients’ obligations, and informed consent	19.805 (<0.001)	3886.0	−3.025 (0.002)	4566.5	−1.140 (0.254)	2920.5	−4.295 (<0.001)
Q17. Professionalism involves respecting patients and colleagues regardless of their gender, background, language, culture, disabilities, and sexual orientation	26.180 (<0.001)	3783.5	−3.204 (0.001)	4286.0	−1.974 (0.048)	2670.0	−4.987 (<0.001)
Q18. Professionalism involves recognizing your own limitations	16.379 (<0.001)	3993.0	−2.677 (0.007)	4422.0	−1.527 (0.127)	3044.5	−3.868 (<0.001)
Q19. Professionalism involves the production and maintenance of accurate patient records	16.724 (<0.001)	3805.5	−3.181 (0.001)	4681.0	−0.729 (0.466)	3071.5	−3.703 (<0.001)
Q20. Professionalism involves considering the patient is the center of care with all interactions, including diagnosis, treatment planning, and treatment, should be aimed to the patient’s best interests	7.199 (0.027)	4352.0	−1.683 (0.092)	4593.5	−0.951 (0.342)	3408.0	−2.669 (0.008)
Q21. Professionalism involves knowing the socioeconomic inequities and inequalities in oral health	5.525 (0.063)	4104.0	−2.286 (0.022)	4341.0	−1.549 (0.121)	4020.5	−0.747 (0.455)
Q22. Professionalism involves that all patients have access to affordable dental treatment	6.864 (0.032)	4583.5	−1.015 (0.310)	3941.5	−2.542 (0.011)	3695.0	−1.664 (0.096)
Ethics and jurisprudence score	12.913 (0.002)	3708.5	−3.137 (0.002)	4702.0	−0.552 (0.581)	3168.5	−3.071 (0.002)
Q23. Professionalism involves establishing a patient–dentist relationship	6.560 (0.038)	4177.5	−2.128 (0.033)	4866.0	−0.157 (0.875)	3516.0	−2.280 (0.023)
Q24. Professionalism involves recognition of patient expectations, desires, needs, and demands during treatment planning and treatment	5.204 (0.074)	4203.0	−2.082 (0.037)	4838.5	−0.235 (0.814)	3674.0	−1.815 (0.069)
Q25. Professionalism involves giving information and professional knowledge to all patients	10.665 (0.005)	3880.5	−2.842 (0.004)	4822.0	−0.270 (0.787)	3320.0	−2.785 (0.005)
Q26. Professionalism involves knowing the intellectual, social–emotional, and language development of children and adolescence	1.380 (0.502)	4525.0	−1.185 (0.236)	4769.0	−0.409 (0.682)	4044.0	−0.686 (0.492)
Q27. Professionalism involves stress management as appropriate	5.212 (0.074)	4136.5	−2.215 (0.027)	4753.0	−0.461 (0.645)	3731.5	−1.608 (0.108)
Q28. Professionalism involves communication with health professionals and can receive and give constructive criticism	6.581 (0.037)	4142.0	−2.218 (0.027)	4794.5	−0.356 (0.722)	3549.0	−2.177 (0.029)
Communication and interpersonal skills score	9.025 (0.011)	3845.0	−2.820 (0.005)	4667.0	−0.645 (0.519)	3463.0	−2.268 (0.023)
Q29. Professionalism include being economically stable, maintaing financial stability or being paid well	20.988 (<0.001)	4489.5	−1.239 (0.215)	3648.5	−3.225 (0.001)	2689.0	−4.484 (<0.001)
Q30. Professionalism include having a good quality of life	5.644 (0.059)	4970.0	−0.014 (0.989)	4108.5	−2.067 (0.039)	3547.0	−2.065 (0.039)
Q31. Professionalism include self‐respect and achieving personal satisfaction	0.039 (0.981)	4960.5	−0.039 (0.969)	4853.5	−0.178 (0.858)	4223.0	−0.159 (0.873)
Q32. Professionalism include balancing work and personal lives	0.234 (0.890)	4832.0	−0.376 (0.707)	4759.5	−0.429 (0.668)	4226.5	−0.150 (0.880)
Quality of life and personal satisfaction score	7.041 (0.030)	4799.5	−0.435 (0.664)	4131.0	−1.969 (0.049)	3339.5	−2.597 (0.009)
Q33. Are you aware of e‐professionalism meaning and aspects?	5.569 (0.062)	4280.5	−1.752 (0.080)	4710.0	−0.536 (0.592)	3472.5	−2.279 (0.023)
Q34. The dentist should communicate with his patients through an account separate from his personal account	3.849 (0.146)	4271.5	−1.866 (0.062)	4832.0	−0.247 (0.805)	3784.5	−1.465 (0.143)
Q35. The dentist should reply to the patients’ questions on the website within his professional knowledge and skills	2.394 (0.302)	4375.5	−1.562 (0.118)	4780.0	−0.376 (0.707)	3937.0	−0.989 (0.322)
Q36. You have to obtain a valid consent from patients to use their comments and photos on the practice’s website	24.183 (<0.001)	3581.5	−3.776 (<0.001)	4706.0	−0.674 (0.500)	2813.0	−4.471 (<0.001)
Q37. Staff members are free to withdraw their permission at any time to upload their photos on the website and they decide which photos are posted of them	4.894 (0.087)	4435.5	−1.372 (0.170)	4567.5	−0.927 (0.354)	3517.5	−2.178 (0.029)
Q38. As a student of dentistry/dentist/specialist, I must keep current on social media use.	14.413 (0.001)	4252.5	−1.836 (0.066)	4081.0	−2.138 (0.033)	2949.5	−3.767 (<0.001)
Q39. The benefits of social media use in dentistry are more than its risks	8.774 (0.012)	4274.5	−1.792 (0.073)	4436.0	−1.262 (0.207)	3254.5	−2.948 (0.003)
Q40. Dentist are responsible to guide patients online in the digital age	2.989 (0.224)	4527.5	−1.143 (0.253)	4692.5	−0.589 (0.556)	3679.5	−1.713 (0.087)
Q41. Patients use social media to get dental information	0.893 (0.640)	4602.0	−0.961 (0.336)	4713.0	−0.541 (0.588)	4166.0	−0.321 (0.748)
Total e‐professionalism score	1.502 (0.472)	4844.0	−0.323 (0.747)	4458.0	−1.147 (0.251)	3944.5	−0.918 (0.359)

*Note: X* ^2^, Chi square statistic at degree of freedom equals to 2; MWU, Mann–Whitney *U* statistic; *Z, Z*‐statistic; *p*, probability value.

Abbreviation: IQR, interquartile range.

^a^Jordanian participants agreed more than did each of the Iraqi and Saudi participants in all significantly different responses and scores.

^b^Saudi participants agreed more than did Iraqi participants in all the significantly different responses except for Questions 29, 30, 33, 38, and 39, as well as in all the significantly different scores except for quality of life and personal satisfaction score.

In addition, no significant differences were identified between genders in the individual item responses, except that females agreed that professionalism should include having the ability to manage a dental practice or patient communication and to oversee financial aspects of practice (Q15, MWU = 7906.0, *Z* = −2.066, and *p* = 0.039), and professionalism should include knowing socioeconomic inequities and inequalities in oral health (Q21, MWU = 7769.5, *Z* = −2.198, and *p* = 0.028). Furthermore, no significant differences were identified between genders in the professionalism total/subscale scores, except that females scored higher on the ethics and jurisprudence professionalism subscale (MWU = 7804.5, *Z* = −2.030, and *p* = 0.042).

Table 5 presents the relationships between the individual professional item responses and the total/subscale professionalism scores and each of the levels of education as well as age. Older participants and participants with higher levels of education agreed more regarding the professionalism of individual items than younger participants and participants with lower levels of education (*p*  < 0.05, Table 5). Additionally, older participants and participants with higher levels of education scored higher on the total professionalism subscale as well as on the ethics and jurisprudence and professional attitude and behavior subscales (*p*  < 0.05, Table 5). Additionally, participants with higher levels of education scored higher on the communication and interpersonal skills subscale (*r* = 0.134 and *p* = 0.022).

**Table 5:** Correlations between individual professional item responses and total/subscale scores and education level and age among the study participants (*n* = 292).

Professionalism items	Spear rho [*r* (*p*)]	
	Education	Age
Total professionalism score	0.121 (0.038)	0.116 (0.048)
Q12	0.143 (0.015)	0.138 (0.019)
Q13	0.150 (0.010)	0.161 (0.006)
Q14	0.075 (0.204)	0.063 (0.280)
Q15	0.120 (0.041)	0.126 (0.031)
Professional attitude and behavior score	0.128 (0.029)	0.129 (0.027)
Q16	0.196 (0.001)	0.171 (0.003)
Q17	0.208 (<0.001)	0.172 (0.003)
Q18	0.174 (0.003)	0.144 (0.013)
Q19	0.166 (0.004)	0.153 (0.009)
Q20	0.189 (0.001)	0.155 (0.008)
Q21	0.098 (0.095)	0.130 (0.027)
Q22	0.032 (0.589)	0.045 (0.441)
Ethics and jurisprudence score	0.174 (0.003)	0.157 (0.007)
Q23	0.130 (0.026)	0.155 (0.008)
Q24	0.161 (0.006)	0.127 (0.030)
Q25	0.154 (0.008)	0.104 (0.075)
Q26	0.059 (0.312)	0.037 (0.532)
Q27	0.092 (0.117)	0.054 (0.357)
Q28	0.168 (0.004)	0.112 (0.056)
Communication and interpersonal skills score	0.134 (0.022)	0.094 (0.108)
Q29	−0.143 (0.014)	−0.033 (0.571)
Q30	−0.018 (0.753)	0.019 (0.747)
Q31	0.097 (0.097)	0.111 (0.058)
Q32	0.057 (0.330)	0.028 (0.634)
Quality of life and personal satisfaction score	−0.026 (0.653)	0.023 (0.692)
Q33	0.025 (0.676)	0.110 (0.060)
Q34	0.125 (0.032)	0.036 (0.535)
Q35	0.052 (0.380)	0.052 (0.372)
Q36	0.166 (0.005)	0.162 (0.005)
Q37	0.183 (0.002)	0.131 (0.026)
Q38	−0.055 (0.345)	0.037 (0.524)
Q39	−0.053 (0.367)	0.052 (0.380)
Q40	−0.029 (0.628)	−0.002 (0.967)
Q41	0.076 (0.195)	0.022 (0.708)
Total e‐professionalism score	0.075 (0.199)	0.100 (0.089)

*Note*: Spearman’s rho, Spearman’s rho correlation test; *r*, correlation coefficient of the Spearman’s rho test; *p*, probability value.

Table 6 presents a comparison of the individual professional item responses and total/subscale professionalism scores between different work sectors among the study population. Significant differences were identified between work sectors regarding the responses to whether professionalism included recognizing patients’ rights, particularly concerning confidentiality, informed consent, and patients’ obligations (Q16, *X* ^2^ = 6.458, df = 2, and *p* = 0.040); professionalism, in which all of the patients had access to affordable dental care (Q22, *X* ^2^ = 8.655, df = 2, and *p* = 0.013); and being aware of the meaning and aspects of e‐professionalism (Q33, *X* ^2^ = 8.757, df = 2, and *p* = 0.013). The participants from the academic sector agreed more than did the participants from both the private and the public sectors in all the significantly different responses (*p*  < 0.05, Table 6). Meanwhile, participants from the private sector agreed more than did participants from the public sector in all significantly different individual item responses.

**Table 6:** Comparison of the individual professional item responses and total/subscale scores between different work sectors of the study population (*n* = 292).

Professionalism items and subscales	Kruskal–Wallis test	Private vs. public^a^		Private vs. academic^b^		Public vs. academic^b^	
	*X* ^2^ (*p*)	MWU	*Z* (*p*)	MWU	*Z* (*p*)	MWU	*Z* (*p*)
Total professionalism score	2.391 (0.303)	1396.0	−0.954 (0.340)	5059.5	−1.558 (0.119)	4246.5	−0.123 (0.902)
Q12	1.999 (0.368)	1481.5	−0.550 (0.583)	5231.5	−1.385 (0.166)	4088.0	−0.587 (0.557)
Q13	2.718 (0.257)	1556.5	−0.026 (0.979)	5265.5	−1.356 (0.175)	3890.0	−1.196 (0.232)
Q14	0.939 (0.625)	1426.0	−0.892 (0.372)	5779.5	−0.087 (0.930)	3975.0	−0.905 (0.365)
Q15	1.063 (0.588)	1459.0	−0.665 (0.506)	5697.5	−0.272 (0.785)	3920.5	−1.035 (0.301)
Professional attitude and behavior score	0.987 (0.610)	1553.5	−0.040 (0.968)	5427.0	−0.842 (0.400)	4024.0	−0.708 (0.479)
Q16	6.458 (0.040)	1482.5	−0.582 (0.561)	4840.0	−2.345 (0.019)	3796.0	−1.427 (0.153)
Q17	2.475 (0.290)	1552.5	−0.052 (0.958)	5231.5	−1.367 (0.172)	3920.0	−1.057 (0.291)
Q18	4.453 (0.108)	1479.0	−0.582 (0.561)	4950.0	−2.039 (0.041)	3938.0	−1.005 (0.315)
Q19	2.489 (0.288)	1552.0	−0.055 (0.956)	5227.5	−1.371 (0.170)	3916.5	−1.062 (0.288)
Q20	1.924 (0.382)	1544.5	−0.105 (0.916)	5286.0	−1.221 (0.222)	3966.5	−0.912 (0.362)
Q21	4.379 (0.112)	1370.0	−1.202 (0.229)	4865.0	−2.086 (0.037)	4104.5	−0.502 (0.616)
Q22	8.655 (0.013)	1157.5	−2.426 (0.015)	5781.5	−0.078 (0.938)	3197.5	−2.846 (0.004)
Ethics and jurisprudence score	2.559 (0.278)	1340.5	−1.288 (0.198)	5088.0	−1.515 (0.130)	4292.0	−0.010 (0.992)
Q23	0.643 (0.725)	1517.0	−0.279 (0.780)	5613.0	−0.460 (0.646)	4021.5	−0.747 (0.455)
Q24	0.828 (0.661)	1491.0	−0.452 (0.651)	5615.0	−0.459 (0.647)	3983.0	−0.858 (0.391)
Q25	1.127 (0.569)	1440.5	−0.747 (0.455)	5340.0	−1.046 (0.295)	4289.0	−0.018 (0.985)
Q26	1.423 (0.491)	1407.5	−0.960 (0.337)	5308.5	−1.124 (0.261)	4240.5	−0.147 (0.883)
Q27	1.014 (0.602)	1532.0	−0.179 (0.858)	5373.5	−0.984 (0.325)	4110.0	−0.497 (0.619)
Q28	3.114 (0.211)	1537.5	−0.148 (0.882)	5108.5	−1.590 (0.112)	3884.5	−1.111 (0.267)
Communication and interpersonal skills score	1.729 (0.421)	1462.0	−0.582 (0.560)	5177.0	−1.338 (0.181)	4150.5	−0.366 (0.714)
Q29	5.871 (0.053)	1169.0	−2.343 (0.019)	5066.0	−1.591 (0.112)	3726.5	−1.448 (0.148)
Q30	2.431 (0.297)	1354.0	−1.241 (0.215)	5126.0	−1.464 (0.143)	4194.0	−0.260 (0.795)
Q31	1.366 (0.505)	1480.0	−0.494 (0.621)	5279.5	−1.166 (0.244)	4135.5	−0.418 (0.676)
Q32	4.284 (0.117)	1453.0	−0.683 (0.495)	5250.5	−1.234 (0.217)	3574.0	−1.900 (0.057)
Quality of life and personal satisfaction score	2.161 (0.339)	1347.5	−1.246 (0.213)	5166.0	−1.348 (0.178)	4204.5	−0.228 (0.820)
Q33	8.757 (0.013)	1425.5	−0.802 (0.423)	4477.5	−2.824 (0.005)	3691.0	−1.544 (0.123)
Q34	0.939 (0.625)	1405.5	−0.971 (0.332)	5501.0	−0.706 (0.480)	4134.0	−0.441 (0.659)
Q35	1.981 (0.371)	1356.5	−1.264 (0.206)	5719.5	−0.213 (0.831)	3799.5	−1.310 (0.190)
Q36	2.884 (0.237)	1495.5	−0.454 (0.650)	5344.0	−1.099 (0.272)	3762.5	−1.498 (0.134)
Q37	0.369 (0.832)	1473.0	−0.527 (0.598)	5771.5	−0.099 (0.921)	4075.5	−0.570 (0.569)
Q38	0.536 (0.765)	1499.5	−0.362 (0.717)	5623.5	−0.411 (0.681)	4034.5	−0.668 (0.504)
Q39	0.001 (0.999)	1554.0	−0.036 (0.971)	5813.0	−0.010 (0.992)	4286.0	−0.026 (0.979)
Q40	1.051 (0.591)	1522.0	−0.230 (0.818)	5494.5	−0.689 (0.491)	3946.0	−0.902 (0.367)
Q41	1.192 (0.551)	1397.0	−0.988 (0.323)	5399.0	−0.899 (0.369)	4135.5	−0.417 (0.677)
Total e‐professionalism score	0.969 (0.616)	1437.5	−0.713 (0.476)	5354.0	−0.953 (0.341)	4241.0	−0.136 (0.891)

*Note: X* ^2^, Chi square statistic at degree of freedom equal to 2; MWU, Mann–Whitney *U* statistic; *Z*, *Z*‐statistic; *p*, probability value.

Abbreviation: IQR, interquartile range.

^a^ = Participants from the private sector agreed more than did participants from the public sector in all significantly different responses.

^b^ = Participants from the academic sector agreed more than did participants from both the private and the public sectors in all significantly different responses.

The stepwise multiple regression analyses showed that older age was associated with higher scores for total professionalism, professional attitude and behavior, ethics and jurisprudence, communication and interpersonal skills, and e‐professionalism (*p*  < 0.05, Table 7). Additionally, higher scores for ethics and jurisprudence were associated with being female, being from a private work sector in comparison to the academic work sector, and being from Iraq (*p*  < 0.05, Table 7). Furthermore, higher scores for quality of life and personal satisfaction were associated with being from Saudi Arabia (*p* = 0.013, Table 7).

**Table 7:** Stepwise linear regression analysis to predict professionalism total and scale scores among the study sample (*n* = 292).

Dependent variable	^a^Predictors	Unstand Co		Stand Co	*t*	*p*	95% CI for *B*	
		*B*	SE	Beta			Lower bound	Upper bound
Total professionalism score (*R* ^2^ = 0.022 and DW = 2.089)	(Constant)	82.105	2.068	‐‐‐	39.695	<0.001	78.034	86.176
	Age	2.594	1.005	0.150	2.580	0.010	0.615	4.572
Professional attitude and behavior score (*R* ^2^ = 0.024 and DW = 2.085)	(Constant)	16.174	0.447	‐‐‐	36.208	<0.001	15.295	17.054
	Age	0.578	0.217	0.154	2.661	0.008	0.150	1.005
Ethics and jurisprudence score (*R* ^2^ = 0.061 and DW = 2.080)	(Constant)	24.085	1.604	‐‐‐	15.014	<0.001	20.928	27.243
	Age	1.249	0.353	0.205	3.538	<0.001	0.554	1.945
	Working in private sector^b^	2.032	0.890	0.132	2.284	0.023	0.281	3.783
	Gender	1.623	0.796	0.117	2.040	0.042	0.057	3.189
Ethics and jurisprudence score (*R* ^2^ = 0.057 and DW = 2.123)	(Constant)	25.184	1.601	—	15.730	<0.001	22.033	28.335
	Age	1.095	0.351	0.180	3.119	0.002	0.404	1.786
	Gender	1.703	0.798	0.123	2.134	0.034	0.132	3.273
	Being an Iraqi^c^	−1.595	0.790	−0.116	−2.017	0.045	−3.150	−0.039
Communication and interpersonal skills score (*R* ^2^ = 0.016 and DW = 2.104)	(Constant)	23.755	0.645	‐‐‐	36.857	<0.001	22.487	25.024
	Age	0.673	0.313	0.125	2.149	0.032	0.057	1.290
Quality of life and personal satisfaction score (*R* ^2^ = 0.021 and DW = 2.196)	(Constant)	15.470	0.292	‐‐‐	52.906	<0.001	14.894	16.046
	Being a Saudi^d^	−1.296	0.521	−0.145	−2.488	0.013	−2.321	−0.271
e‐Professionalism score (*R* ^2^ = 0.016 and DW = 2.206)	(Constant)	32.192	0.815	‐‐‐	39.481	<0.001	30.588	33.797
	Age	0.858	0.396	0.126	2.165	0.031	0.078	1.638

*Note: R* ^2^, coefficient of determination; DW, Durbin Watson test statistic; *B*, beta statistics; *t*, *t* statistics; *p*, two‐tailed probability value.

Abbreviations: CI, confidence intervals; SE, standard error; Stand Co, standardized coefficient; Unstand Co, unstandardized coefficient.

^a^In all models, the vertical inflation factor (VIF) value was 1.

^b^Saudi and Jordan were the reference categories in the relevant models.

^c^Academic work sector is the reference category in this model.

^d^Iraq and Jordan were the reference categories in the relevant models.

## 4. Discussion

Current outcomes highlighted significant differences in awareness and attitudes toward professionalism across participants from Jordan, Iraq, and Saudi Arabia. Among those, Jordanian and Saudi participants exhibited greater awareness of the meaning and aspects of professionalism than did the participants from Iraq. Similarly, previous studies have indicated regional variations in the understanding and application of professionalism in healthcare and education settings [26, 27]. For instance, it was found that medical students in Saudi Arabia had a well‐developed understanding of professional values, which explained the higher awareness observed in the current study [28]. Similarly, one more study emphasized the role of curricular interventions in enhancing professionalism among medical students in the Middle East [29]. Educational reforms in Jordan and Saudi Arabia have placed a stronger emphasis on integrating professionalism into medical and dental education [30, 31]. It was also reported that curricular changes in Jordanian medical schools led to improved student attitudes toward professionalism [32]. On the other hand, Saudi Arabian dental students recognized the importance of professional education, which reflects the country’s commitment to enhancing professional standards in healthcare [33].

Remarkably, the study outcomes further revealed that more participants from Saudi Arabia than from Jordan or Iraq had received educational or training courses about professionalism. This finding is consistent with previous efforts, which highlighted initiatives in Saudi Arabia to incorporate professional training in healthcare education [34]. It was also noted that structured professionalism courses in Saudi medical schools contributed to a better understanding and application of professional principles among students [35]. Interestingly, the current outcomes showed that the willingness to take courses on professionalism did not significantly differ among participants from the three countries, indicating a generally high interest in further education on the subject. This outcome is encouraging and suggests the universal recognition of the value of professional training across different educational contexts [36]. Prior inquiries have also emphasized the importance of continuous professional development and lifelong learning in maintaining and enhancing professional standards [37], and the findings of the present study support this perspective, which indicates a broad‐based willingness among dental students and professionals to engage in ongoing professional education [38].

Across countries, Jordan and Iraq have slightly higher e‐professionalism scores than does Saudi Arabia, and previous research also indicates that different educational and professional standards in these countries influence e‐professionalism [39]. They highlighted that e‐professionalism training is more emphasized in Jordanian and Iraqi curricula, potentially explaining the higher scores observed in these countries [40]. Similarly, females scored higher on e‐professionalism than males did. This shows that female professionals often exhibit a higher level of professionalism, with cultural and societal expectations expected to contribute to these gender differences in professionalism [41]. In terms of the work sector, private sector professionals had slightly higher e‐professionalism scores than did those in the public and academic sectors. This observation highlighted that private sector employees often have access to more resources and training opportunities that enhance their professional skills [42].

Current outcomes indicated that professional attitude and behavior subscale scores did not differ significantly across countries, genders, or work sectors. This equality suggests a relatively consistent baseline level of professional attitude and behavior [43], irrespective of these variables, because foundational professional attitudes and behaviors are universally emphasized across different educational and professional settings [44]. Similarly, in terms of the current outcomes, the Ethics and Jurisprudence scores were slightly greater in Saudi Arabia than in Jordan and Iraq. Several researchers have also reported that recent reforms in Saudi Arabia’s healthcare education have placed a stronger emphasis on ethics and jurisprudence [45]. They particularly noted that these reforms have led to enhanced ethical training and awareness among Saudi healthcare professionals [46]. Interestingly, females again scored higher than males on this subscale, supporting the notion that female professionals might be more attuned to ethical considerations, ethical perceptions, and behaviors, which stem from differing socialization processes and expectations [47, 48]. Additionally, the current findings also highlighted that professionals in the private sector scored higher on ethics and jurisprudence than did those in the public and academic sectors. This tendency was also observed, and it was argued that the competitive nature of the private sector often drives employees to adhere more strictly to ethical standards to maintain their professional standing [49].

Across the study variables, the communication and interpersonal skills scores showed minimal differences, which indicated a generally high level of competence in this area among the participants. It was reported that communication skills are a critical focus in medical and dental education across the Middle East [50], resulting in uniformly high proficiency levels [51]. Conversely, quality of life and personal satisfaction scores were slightly greater in Iraq and Jordan than in Saudi Arabia because of differences in work–life balance and job satisfaction reported in these countries [52, 53]. Their studies specified that professionals in Jordan and Iraq experienced greater personal satisfaction due to different work environments and cultural expectations [54]. Among those, it was also observed that females reported higher satisfaction scores than males did, which aligns with previous findings [55], suggesting that women prioritized personal satisfaction and work–life balance more than men did. Additionally, professionals in the private sector reported higher satisfaction levels than did those in the public and academic sectors [56], supporting the observations regarding the benefits of private sector employment [57].

In Jordan’s participants, the overall professionalism scores were the highest, followed by those in Saudi Arabia and Iraq, because of the strong professionalism training programs in Jordan [58]. Excitingly, females again scored higher than males, reinforcing the earlier trend of greater professionalism among female professionals [59]. In addition, in terms of the work sector, the highest scores were observed in the private sector, followed by the public and academic sectors. Private sector employees often receive more comprehensive training and development opportunities that enhance their professionalism [60].

The overall professionalism score seems to vary significantly among Jordanian, Iraqi, and Saudi participants, with Jordanians rating their professionalism higher than Iraqis but not significantly different from Saudis. These outcomes indicated that cultural and educational backgrounds influence perceptions of professionalism [61], and it was also found that medical students from different countries had varied understandings of professionalism based on their training environments and the role of cultural context in shaping professional values and behaviors [17, 62]. Similarly, the scores for professional attitude and behavior also showed significant variation among the countries. Jordanians scored higher than Iraqis did, with no significant difference between Jordanians and Saudis. It has been noted that professional attitudes are heavily influenced by educational settings and cultural norms [62]. It was also emphasized that the hidden curriculum in medical education profoundly impacts students’ professional development, suggesting that Jordan’s educational approach fosters stronger professional attitudes [63].

The current study revealed that participants from Jordan had much greater scores in ethics and jurisprudence than participants from Saudi Arabia and Iraq because this difference in curriculum emphasis on ethical and legal concerns among the three nations is attributed to these variances in ethics and legal issues [64]. Additionally, the communication and interpersonal skills scores also differed significantly, with Jordanians scoring higher than both Iraqi and Saudi participants, which is attributed to the importance of communication skills training in medical education [65]. Additionally, the higher scores among Jordanians suggest a stronger emphasis on these skills within their educational programs, which is why there are more training centers in Jordan [65]. In addition, there were significant differences in quality of life and personal satisfaction scores, with Jordanian participants reporting higher satisfaction than Iraqi and Saudi participants, aligning with prior findings that work–life balance and economic stability are important for professionals’ quality of life [66]. Although the total e‐professionalism scores did not differ significantly among the countries, specific items related to social media use in dentistry and guiding patients online showed variation, which indicated that digital literacy and cultural attitudes towards social media significantly influence e‐professionalism practices [67], suggesting varying levels of engagement and attitudes towards social media use in professional contexts across the countries [68].

According to the findings, the correlations between the scores on the professionalism items and demographic factors such as education level and age were significant. The total professionalism score positively correlated with both education level and age, suggesting that higher education levels and older age enhance professional attitudes and behaviors in dental students [69]. This finding is also consistent with research indicating that educational attainment has a positive influence on professional conduct [70]. The professional attitude and behavior score also showed positive correlations with both education and age, particularly in recognizing patients’ rights regarding confidentiality and informed consent, highlighting that higher education levels enhanced ethical understanding and implementation [70]. In a similar vein, the positive correlation of ethics and the jurisprudence score with education and age highlights education’s role in promoting adherence to ethical and legal standards [71]. Additionally, the communication and interpersonal skills scores correlated significantly with education level but not age, indicating that educational experiences are central to developing communication skills [72]. Conversely, the quality of life and personal satisfaction scores did not significantly correlate with education level or age, suggesting that these aspects of professionalism are influenced by a broader range of factors beyond education and age, as noted in previous studies [73].

According to the results of the present study, the comparison of the responses to the items related to professionalism and the total/subscale scores between different work sectors revealed notable differences. In this regard, the overall professionalism score did not show significant variation across work sectors, supporting the idea that professionalism is a fundamental value that is consistently upheld across various dental practice environments [74]. For some specific items, the recognition of patients’ rights, particularly concerning confidentiality and informed consent, was significantly greater among participants from the academic sector than among those from the private sector. This result aligns with research that highlights the strong emphasis on ethical principles within academic settings [75]. Additionally, recognizing one’s own limitations demonstrated significant differences, with academic sector participants scoring higher than their private sector counterparts due to the structured feedback and self‐reflection practices prevalent in academic environments [76]. The item related to ensuring that all patients have access to affordable dental care showed significant differences across sectors, with private sector participants agreeing more than public sector participants. These outcomes also support previous findings that public sector professionals often face more significant challenges in providing affordable care due to systemic constraints [77]. Regarding e‐professionalism, significant differences were observed in awareness and understanding and the obligation to stay current on social media use, with higher scores among academic sector participants. This observation suggests that academic institutions are more proactive in incorporating e‐professionalism into their training programs and reflecting trends in broader healthcare education [78].

Moreover, stepwise linear regression analysis revealed that age, work sector, gender, and nationality significantly influence professionalism among dental professionals. Age positively predicted the total professionalism score, accounting for 2.2% of the variance (*R* ^2^ = 0.022), consistent with studies indicating that professionalism increases with age and experience, as older professionals have more time to internalize professional values [79]. Similarly, age explained 2.4% of the variance (*R* ^2^ = 0.024) in the professional attitude and behavior score, supporting findings that older professionals exhibit greater professionalism due to a more developed sense of responsibility and ethical standards [80]. According to the ethics and jurisprudence scores, age, work sector, and gender accounted for 6.1% of the variance (*R* ^2^ = 0.061). Therefore, older professionals and those in the private sector scored higher, reflecting increased ethical training and accountability mechanisms present in the private sector [80]. In addition, the literature indicates that gender differences in ethical perceptions and behaviors lead to variations in scores, with some studies suggesting that males and females prioritize different ethical principles in their conduct [81]. Likewise, age explained 1.6% of the variance (*R* ^2^ = 0.016) in communication and interpersonal skills, indicating that these skills improve with experience, which is necessary for effective patient interactions, and the professional relationships literature also supports that communication skills are honed over time and through practical experience [82]. Additionally, nationality significantly influenced quality of life and personal satisfaction, accounting for 2.1% of the variance (*R* = 0.021), highlighting the impact of sociocultural and economic factors on satisfaction [83]. Several studies have revealed that cultural context significantly affects life satisfaction, as societal norms and expectations shape individuals’ perceptions of their personal and professional lives [83]. Correspondingly, age also predicted the e‐professionalism score, explaining 1.6% of the variance (*R* ^2^ = 0.016), suggesting that older professionals have a greater awareness of the risks and responsibilities associated with digital professionalism because digital professionalism requires ongoing education and adaptation, particularly for younger professionals, who are more accustomed and habituated to informal online interactions [84].

### 4.1. Strengths, Limitations, and Recommendations

The use of stepwise linear regression to identify key predictors of professionalism provided a detailed understanding of how various factors influence professional behavior. Likewise, the comparative approach of examining participants from three different countries also offered insights into regional variations in professionalism and contributed to the broader literature on cultural impacts in healthcare education. Likewise, the inclusion of participants from different work sectors and educational backgrounds also ensured the broad applicability of the findings across various professional environments. Despite these strengths, the study also has several limitations. For instance, the cross‐sectional design does not capture changes in professionalism over time, which makes it difficult to assess the long‐term impact of educational and training interventions.

Therefore, to enhance professional training, countries, particularly Iraq, should integrate more comprehensive professional training into their dental and medical curricula to bridge the gap observed in professional awareness and attitudes. Then, they must encourage ongoing professional development and lifelong learning to maintain and enhance professional standards, with a particular focus on e‐professionalism. Furthermore, researchers should develop targeted interventions that address gender differences in professional attitudes and behaviors, ensuring that both male and female professionals receive adequate support and training. They must tailor professional training and development programs to the specific needs of the private, public, and academic sectors, recognizing the unique challenges and resources of each while considering cultural norms and expectations, which have been identified as shaping factors of professional values and behaviors. Therefore, these kinds of longitudinal studies will help to better understand how professionalism develops over time and the long‐term impact of educational reforms and training programs. The results of the present study may help scholars perform comparative studies involving additional countries to identify global versus regional influences on professionalism in healthcare. Finally, to explore further influencing factors, researchers should further apply qualitative methods to gain deeper insights into the contextual factors and personal experiences that shape professionalism among dental professionals.

There were significant variations in professionalism awareness and e‐professionalism observed across the three countries investigated in this study. These variations highlight the importance of developing dental education curricula that are culturally sensitive. For example, dental schools in Iraq might need more structured professionalism education compared to Jordan and Saudi Arabia, where such curricula have already been implemented. Dental schools should consider these findings to establish minimum standards in professionalism education, including e‐professionalism, and to prepare students to meet ethical, communication, and professional standards in their practice. These steps will promote individual professional growth and, in turn, enhance patient trust and the quality of care within the dental practice.

This study provides valuable insights into dental professionalism across multiple countries; however, it has some limitations, including: First, the cross‐sectional design restricts our ability to infer causality or examine changes in professionalism over time. Thus, longitudinal studies are needed to assess how professionalism develops throughout dental education and clinical practice. Second, the reliance on self‐reported questionnaire data introduces the potential for response bias and social desirability bias, which may have influenced participants’ responses. It is recommended to incorporate objective measures or third‐party assessments to overcome these biases. Third, the unequal sample sizes between Jordan, Iraq, and Saudi Arabia may have affected the comparability of results and the generalizability of country‐specific findings. Therefore, using a larger sample size is recommended in future research. Finally, using quantitative methods alone has limited the ability to find out the contextual and experiential factors that contribute to professionalism. Using qualitative approaches, such as interviews or focus groups, would provide a better picture to explain the observed differences and give more tailored educational interventions.

## 5. Conclusions

Within the limitations of the current study, the following conclusions can be drawn. Regional differences in awareness and attitudes toward professionalism among dental professionals and students from Jordan, Iraq, and Saudi Arabia were highlighted, and notably, Jordanian and Saudi participants demonstrated higher levels of professionalism than did participants from Iraq. Factors such as age, gender, work sector, and nationality significantly influenced professionalism, with older professionals, females, and those in the private sector generally scoring higher. The study also highlighted uniformly high communication skills across regions and variations in e‐professionalism scores. It was also emphasized that sociocultural factors contributed to improved quality of life and personal satisfaction in Jordan and Iraq.

## Funding

No funding was received for this manuscript.

## Conflicts of Interest

The authors declare no conflicts of interest.

## Data Availability

The data that support the findings of this study are available from the corresponding author upon reasonable request.
